# Two-Stage Shape Memory Alloy Identification Based on the Hammerstein–Wiener Model

**DOI:** 10.3389/frobt.2019.00083

**Published:** 2019-09-04

**Authors:** Dorin Copaci, Luis Moreno, Dolores Blanco

**Affiliations:** Department of Systems Engineering and Automation, Carlos III University of Madrid, Leganes, Spain

**Keywords:** shape memory alloy, modeling, Hammerstein–Wiener, control, actuator

## Abstract

Thanks to characteristics, such as high force and light weight, a good biocompatibility, noiseless operation and simplicity, and relatively low-cost compared with other conventional actuators, actuators based on shape memory alloy are currently one of the most interesting research topics. They have been introduced in applications such robotics, medicine, automation, and so on. For a good actuator integration of these types of applications, proper control is needed, which seems to be a difficult task due to the hysteresis, dilatory response, and non-linear behavior. This work presents a new form of modeling of this type of actuator based on Hammerstein–Wiener model. This has been identified in two stages of the operation. When the activation temperature for the actuator is obtained by the Joule effect, electrically energy is transformed into thermal energy. In the second stage, the thermal energy is transformed into mechanical work. To fulfill this objective, experimental data [e.g., the input signal (pulse-width modulation), temperature signal, and position signal] from the two stages was obtained for a specific shape memory alloy wire and for specific environmental conditions. This data was used in the modeling process. The final model consists of a combination of the models from the two stages, which represent the behavior of the shape memory alloy actuator where the input signal is the pulse-width modulation signal and the output signal are the position of the actuator. Our results indicate that our model has a very similar response to the behavior of the real actuator. This model can be used to tune different control algorithms, simulate the entry system before manufacture and test on real devices.

## 1. Introduction

In recent years, Shape Memory Alloys (SMAs) materials have been considered to be a promising technology for the development of non-conventional actuators oriented on some specific applications. SMA-based actuators have characteristics which make them suitable to be integrated into a large number of applications. In particular, they have a high force-to-weight ratio, noiseless operation, present a low volume (SMAs can generate about 150 times higher force compared with the hydraulic actuators and 400 times higher force compared with the magnetic actuators, at the same volume), and are relative low-cost solution compared with other actuators. In the last few years, SMAs have been used in a wide variety of applications (Jani et al., 2014), among them: aerospace (Chau et al., 2006; Hartl and Lagoudas, 2007); automotive industry (Stoeckel, 1990; Butera et al., 2007); medical applications (Morgan, 2004); and robotics (Sreekumar et al., 2007). Also, because of their flexibility, they can be considered to be a good actuation solution for soft robotics applications and especially for rehabilitation devices. They can be considered to be an alternative to conventional actuators, such DC and AC motors for robotics applications where a high force at low velocity is needed; or to the pneumatic muscles if a low weight, low size and noiseless operation is required.

SMA is a smart material that has the capacity to remember a previous shape that was defined after the material was subjected to a suitable thermomechanical procedure. This effect is called “shape memory effect” (SME). This type of material has two phases: a low-temperature phase, which is called martensite; and a high-temperature phase, which is called austenite. Between these two phases, the material can be deformed up to 10% of his total length, this effect is known as “superelasticity” (SE). In the majority of applications where this type of material is found, the SE effect is used. The SMAs have a non-linear behavior with a large hysteresis, which makes them difficult to model and control. The most common alloy is Ni–Ti, or Nitinol (Dynalloy, 2018), but alloys of Nitinol with other metals can also be found (Saes, 2018). When the SMA is proposed to be used in as actuator in any robotic application, important factors must to be considered to select the most suitable configuration:

- The shape of SMA. The SMA can be wire, spring, ribbon and diaphragm, tubing.
- The temperature of activation (temperature of austenite phase). This can depending on the alloys but in robotics applications the most common are the SMA activated in 90°C.
- The force that can be achieved.
- The technique to heat and cool them, which depends on the thickness of the alloy.
- The adopted control technique.

To extend the use of these materials as soft actuators, it is essential to develop appropriate mechanical design and suitable control strategies to improve the operational frequency, bandwidth and the durability and reliability of SMA actuators. It is essential to improve the control technique and the first step to develop suitable control strategies is SMA material modeling.

To apply a classical model-based control approach for an SMA actuator, highly complex and non-linear models are necessary. A model of the material will allow us to simulate the behavior of different control algorithms to analyse their performance. Control algorithms can be redesigned and tested before they are implemented in real equipment. Meanwhile, the analysis of the system behavior on the simulation can avoid any damage that may be occurred if the control algorithm is implemented directly in the real system. Consequently, one of the important fields in robotics is to obtain a model of the real system, which offers the possibility to simulate the entire system.

In the last decade, several studies have been conducted on the different approaches to model or estimate the behavior of the SMAs. Much of the current work was centered in the modeling of hysteresis behavior. In Song et al. (2003), the hysteresis behavior of a SMA is modeled with a neural network, which is used such an inverse model in the control algorithm. In Hughes and Wen (1997), a Preisach model was used to describe the hysteresis behavior of SMA and piezoceramics actuators and in Mart́ın Clemente (2013) Bouc-Wen model and Prandtl–Ishlinskii model was evaluated to describe this behavior. Another approach is based on the modeling of the electrical resistance, as proposed in Novák et al. (2008) and Cui et al. (2010). Thermal models, such as Iadicola and Shaw (2004), simulate the inhomogeneous nature and the thermo-mechanical coupling of stress-induced transformation seen in shape memory alloy and Velázquez and Pissaloux (2012), where the thermal behavior model of Nickel-Titanium (Ni–Ti) was developed and used to improve the actuation speed.

The working principle of this type of actuator is based on the heating effect. One of the most common methods to heat SMA wires to obtain the desired displacement and force based on SE effect is to heat by means of the Joule effect. In this process, two transduction subprocesses take place: first, electric energy is transformed into thermal energy by the Joule effect; and second, the thermal energy is transformed into mechanical work. A new approach to modeling the SMA wire is introduced in this work, which is based on two transduction subprocesses: first, a model capable to simulate the behavior transformation from electrical energy to thermal energy was identified; and second, a model was designed to simulate the thermal energy to mechanical work transformation. The combination of these two models represents the process transformation: electrical energy - thermal energy - mechanical work, which simulates the behavior of the SMA wire.

This paper is divided into five sections. Section 2 presents the methodology. It presents the SMA wire characteristics, the test bench setup used in this work and the hardware. Section 3 gives the details of the strategy adopted to model the SMA wire. Sections 4 and 5 present the preliminary results and offer some conclusions.

## 2. Methodology

For the SMA wire model, it is necessary to identify the input/output signals. For the SMA wire contraction movement (heating stage), the input and output signals are saved. To obtain a measurement of the input/output signals, a test bench has been used where the SMA wire is heated by the Joule effect and its position is measured. This section presents the characteristics of the wire used in the identification process, the test bench where the experimental work was made, and the electronics hardware used during this work.

### 2.1. SMA Wire Characteristics

Currently, there is a large variety of SMA wires in the market, with different characteristics, such as: Ni–Ti, Ni–Ti with different alloys and different diameters. Depending on the alloy, the SMA wires presents different characteristics with regard to the activation temperature, force and stroke. A specific type of SMA wire of Flexinol® (Dynalloy, 2018) has been used in this work to find its model and to check the result of the model identification.

There is a relationship between the diameter of the SMA wire of Flexinol® (Dynalloy, 2018), the force, and the cooling time. In Table 1, the first column presents the diameter of the wire, the second column presents that actuation force, which guarantees a lifetime of tens of millions of cycles, and the last two columns present the cooling time for two types of wires, with activation temperatures of 70 and 90°C, respectively. According to the table and the objectives of our research group [development of soft rehabilitation devices such elbow wearable exoskeleton, as presented in Copaci et al. (2017)], it has been decided to work with 0.51 mm wires activated at 90°C because the maximum force is obtained with this diameter and the cooling time (back to the ambient temperature when the SMA wire returns to the martensite phase) is lower when compared to the wire activated at 70°C.

**Table 1:** Properties of the SMA wires (Dynalloy, 2018).

**Diameter**	**Force**	**Cooling time**	**Cooling time**
**size [mm]**	**[N]**	**70^**°**^C [s]**	**90^**°**^C [s]**
0.025	0.0089	0.18	0.15
0.038	0.02	0.24	0.2
0.050	0.36	0.4	0.3
0.076	0.80	0.8	0.7
0.100	1.43	1.1	0.9
0.130	2.23	1.6	1.4
0.150	3.21	2.0	1.7
0.200	5.70	3.2	2.7
0.250	8.91	5.4	4.5
0.310	12.80	8.1	6.8
0.380	22.50	10.5	8.8
0.510	35.60	16.8	14.0

The characteristics of the wire used in this work are: the diameter of wire is 0.51 mm, the activation temperature is 90°C, the length of wire is 230 mm with a nominal force of 35.6 N from Dynalloy (2018) company. The ambient temperature when the experimental test was done at around 23°C. To activate the SMA wire with the Joule effect, a current of maximum 4A was applied. The total stroke of this wire is between 3 and 5 %, according to the attached weight (if is attached to a spring is 3% of stroke and if it is a dead weight this is 4%). In this work, we considered a stroke of 4%.

### 2.2. Test Bench

There are no standardized methods to characterize the behavior of SMA wires. Consequently, our research group at the Carlos III University of Madrid has designed and built a test bench that has allowed us to obtain accurate experimentally measured values for model identification.

**Figure 1:**
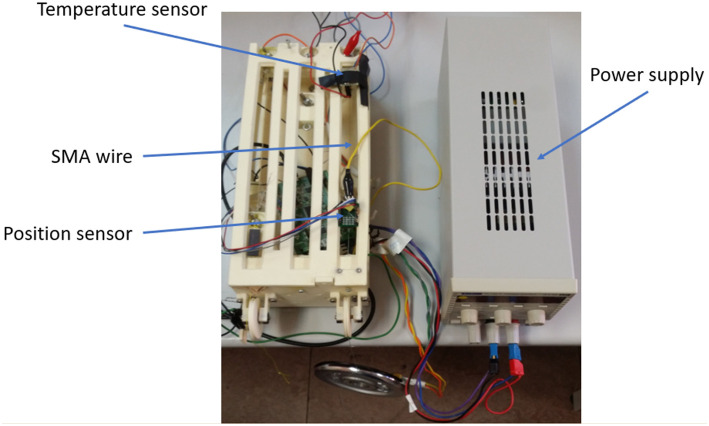
Test bench for SMA actuators.

The test bench permits us to attach three SMA wires with a length up to 0.27 m, which can displace a variable or a constant charge up to 1 kg. This presents three independently rails, which can be used to test three actuators of SMA. One of the ends of the SMA wire is fixed to the structure of the work bench. The non-fixed end is crimped to a movable part. When the wire contracts, the movable part is displaced and the position sensor measures the displacement of the non-fixed part. The test bench charged with 1kg can be seen in Figure 1.

### 2.3. Electronics Hardware

The electronic hardware comprises the power electronics, the controller, and the sensors that are used in the test bench. The power electronics, which are used to activate the SMA wire, are based on a MOSFET transistor (STMicroelectronics STP310N10F7), which works as a commutation circuit and amplifies the control signal, pulse width modulation (PWM) generated by the controller. The power electronics are connected to the extremity of the SMA wires.

The controller is a 32-bit microcontroller STM32F407G from STMicroelectronics®, which can be fully programmed with Matlab/Simulink® (Caballero et al., 2016).

With these electronics, the control hardware architecture is able to manage three distinct SMA wires (the number of rails available on the test bench).

The test bench presents two types of sensors: NSE–5310, 12–bit linear position sensor from amsAG company (amsAG, 2019) and MLX90614 infrared thermometer for non-contact temperature measurements from Melexis company (Melexis, 2019). The position sensor is based on a Hall effect sensor and is composed of two pieces: the sensor and a magnetic strip, which is placed in the movable part of the test bench. The infrared thermometer is capable of measuring a temperature range between −40°C and 125°C.

## 3. SMA Modeling

According to the characteristics of the SMA material—in the contraction stage (heating stage), the actuator requires the input signal, represented by the power supply signal; and in the recovery stage (cooling stage), the actuator does not present the input signal and the SMA wire behavior depends instead on the recuperation force, the diameter of the wires and the ambient temperature (Copaci et al., 2015). Due to the absence of the control signal in the cooling stage, it was not possible to model the SMA wire in this phase. Consequently, the SMA model is only reliable in the heating stage.

**Figure 2:**
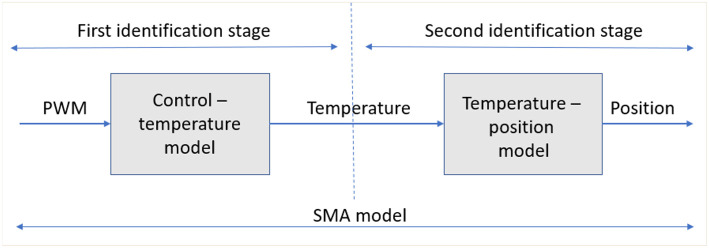
SMA identification stages: first stage control-temperature model and second stage temperature position stage.

In the contraction stage (heating stage), the SMA wire passes through two transformation subprocesses: in the first, the electrical energy is transformed into thermal energy; and in the second, the thermal energy is transformed in mechanical work. In the cooling stage, the SMA wire recuperates the initial form over the recuperation force (the weight attached to the movable part of the test bench), wire diameter and ambient temperature, and does not present an input signal from the controller. In the heating stage, due to the existence of the two subprocesses, this work proposes an identification of SMA wire with two black box models corresponding to these two subprocess. The final SMA wire model is represented by a combination of these two models in series (Figure 2).

The estimated SMA wire model (combination of the electrical energy–thermal energy model and thermal energy–mechanical work) have been done according to the input/output signals, using non-linear models based on the Hammerstein–Wiener model (Lennart, 1999).

### 3.1. Hammerstein–Wiener Model

The Hammerstein model, or a combination of Hammerstein's and Wiener's models, have proven to be able to accurate when used to describe a wide variety of non-linear systems, such as chemical process (Eskinat et al., 1991), electrically stimulated muscle (Hunt et al., 1998; Bai et al., 2009; Le et al., 2012), electrical drivers (Balestrino et al., 2001), biological systems (Hunter and Korenberg, 1986), magneto–rheological dampers (Wang et al., 2007), ultrasonic motors (Zhang and Tan, 2008; Copaci et al., 2014), thermal process (Sung, 2002).

**Figure 3:**
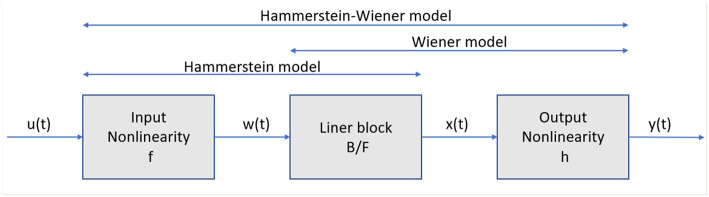
Structure of the Hammerstein–Wiener model.

The Hammerstein model was first presented in Narendra and Gallman (1966) and the Wiener model has roots in a non-linear system using Voltera expansions (Wiener, 1942). The Hammerstein–Wiener model is practically is composed of a non-linear–linear block (Hammerstein model) followed by a linear–non-linear block (Wiener model). This model is based on a combination of a linear and non-linear systems with a transparent relationship between them, which makes it easier to implement than other non-linear methods. This model is composed by one linear block (a discrete transfer function) and one or two non-linear blocks (see Figure 3) (Matlab, 2019).

In Figure 3, the structure of the Hammerstein–Wiener model is composed of two non-linear blocks and one linear block. The first block represented by *w*(*t*) = *f*(*u*(*t*)), is computed from the input data. This term represents an input non-linearity, a static function, where the value given in time *t* depends on the input value in time *t*. The second block represents a linear transfer function *x*(*t*) = (*B*/*F*)*w*(*t*). In this case, the transfer function (*B*/*F*) can be configured by specifying the order of the numerator B, as well as the order of the denominator F. The non-linear output block is represented using a non-linear function *y*(*t*) = *h*(*x*(*t*)). Like the input non-linear function, the output non-linear function is a static function.

### 3.2. Hammerstein–Wiener Model for SMA Wire

To obtain an accuracy SMA wire identification based on Hammerstein–Wiener model the input/output data of the two subprocesses was saved. To obtain all necessary data for the identification process, a data acquisition program was built in Matlab/Simulink. The necessary data, PWM signal, temperature, and position was saved with a frequency of 500 Hz. For system modeling the System Identification Toolbox from Matlab/Simulink (Matlab, 2019) was used.

**Figure 4:**
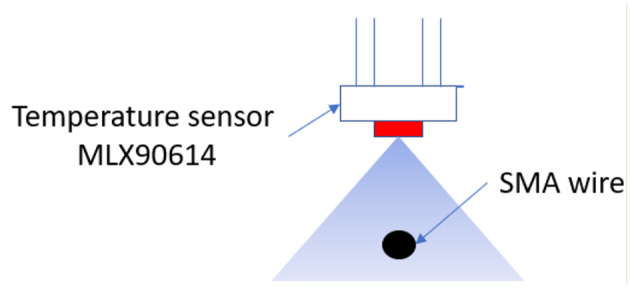
MLX90614 temperature setup over the SMA wire.

For the first subprocess the input data is represented by the PWM signal which is generated by the controller to govern the power electronic stage and the output signal is the temperature of the wire. Due to the temperature sensor setup in the test bench (Figure 4), which is not in contact with the SMA wire, the measured temperature is an average between SMA wire temperature and the temperature of the ambient around the SMA wire. This average was used in the identification process, although a more accurate temperature, only from the SMA wire, permits a better model estimation. In Figure 4, the blue-triangle zone represents the area of measurement of the MLX90614 sensor.

**Figure 5:**
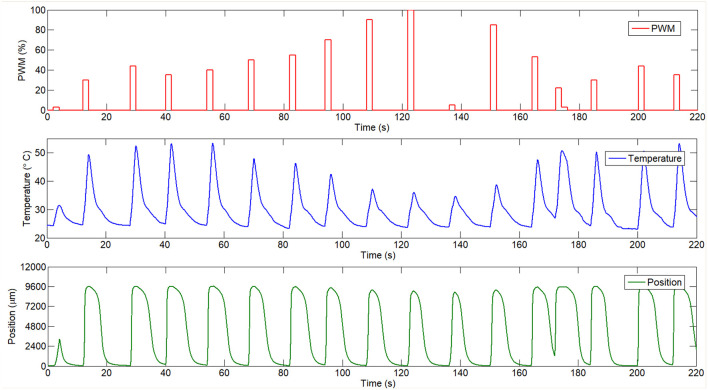
Input and output signals used in identification process.

First, the input signal which consist in PWM signals with different amplitude was saved and imported to the program. This signal can be seen in the Figure 5. The temperature measured with the sensor is used like an output signal and in the second model identification (temperature–position) it is used like an input signal. Finally, the output signal for the second model is represented by the final position (wire contraction). These signals can all be seen in Figure 5.

The process of the models identification is based on the test–error, similar with the scheme presented by the Lennart (1999) (System identification loop). According to the SMA wire behavior (presenting non-linearities) different non-linear models from System identification toolbox of Matlab, such as NARX and Hammerstein–Wiener have been pre–tested. According to the firstly results and the possibility to configure each block (non-linear, linear, and non-linear) a Hammerstein–Wiener model with one input and one output was used for each model. In the model PWM–temperature the block of Hammerstein–Wiener was configured: the linear block was configured like an order three transfer function with three poles and two zeros. The non-linearity blocks were configured with one dimensional polynomial with two units for the input non-linearity and such a piecewise linear with 10 units for the output non-linearity. For the second model, temperature–position the linear block is a three-order transfer function with three poles and two zeros and the non-linearity blocks are a wavelet network with 88 units for the input non-linearity and a sigmoid network with 10 units for the output non-linearity. The order of the linear transfer function and the non-linearity input/output signal were chosen experimentally and considering the shape and behavior of the signals. The lineal transfer function order has been chosen according with the SMA behavior in the open loop and based on a test–error algorithm trying firstly with a second order after third. Similar, the non-linear blocks are configured with the test–error algorithm after each change validating the model and analysing the results.

**Figure 6:**
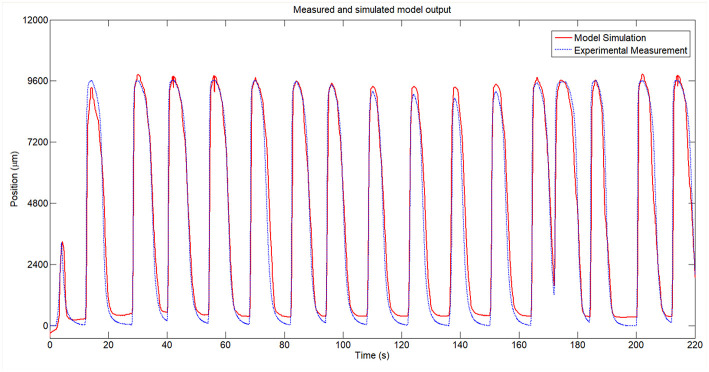
Measured and simulated model of the SMA wire.

After the identification, we obtained a 92.38% similitude between the estimated model and the measured temperature and 80.42% similitude between the estimated position model and the measured SMA wire position. The identification results can be seen in the Figure 6, where the output signal for the two models in series (position) is compared with the measured signal for the real SMA wire.

Due to the absence of the control signal in the cooling stage, it was not possible to model the SMA wire (with the same technique in the case of PWM-temperature model due to the fault of PWM signal) in this phase and the SMA model of the heating stage was used in the two phases (heating and cooling). Thus, the SMA model is only reliable in the heating stage. The cooling stage model can be obtained for temperature- position or can be identified with different tools such curve fitting. In this study, the SMA model is used to control algorithm calibration which is used heating stage. The actuator recuperation (cooling stage) depend of the ambient temperature and the control signal in this case is 0. In Figure 6, is presented the responses of the simulated model and the real SMA wire.

**Figure 7:**
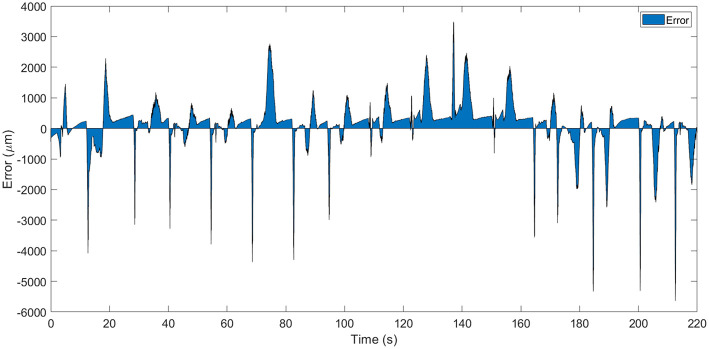
Error area between simulated model and real SMA wire.

**Figure 8:**
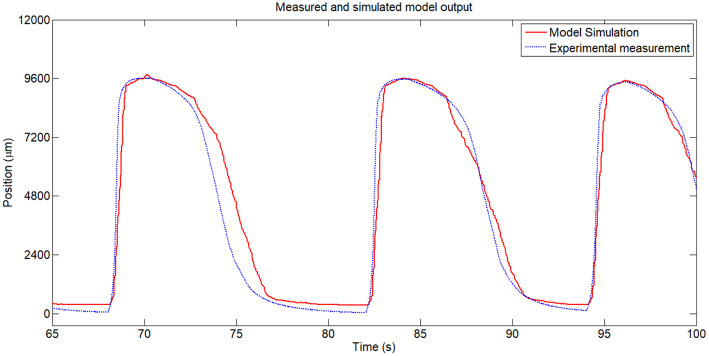
Zoomed area from Figure 6.

A zoomed area from the Figure 6 between the *t* = 65*s* and *t* = 100*s* can be seen in Figure 8 and the error area can be seen in Figure 7. From these figures can be seen that in open loop, in the heating stage the maximum error between the identified SMA model and the real SMA behavior is around 3 mm (in the firstly cycles) but in the cooling stage this error increase to 5 mm. However, the similarity of the model with the real SMA behavior permits to calibrate the control algorithms.

## 4. Results

To evaluate the model performances, a PID controller was tuned over the SMA wire model and the same controller was tested in parallel on the test bench. The PID controller is used to send a PWM current *C*(*z*) to the actuator according to the following (Equation 1):

(1) $$\begin{array}{r} C\left(z\right)=\left[K_{p}+\frac{K_{i}}{1-z^{-1}}+K_{d}\left(1-z^{-1}\right)\right]E\left(z\right), \end{array}$$

where *C*(*z*) is the PWM duty cycle, *K*_*p*_ is the proportional gain, *K*_*d*_ is the derivative gain and *K*_*i*_ is the integral gain. *E*(*z*) is the error between the reference and the output signal (provided by the position sensors).

The PID controller was tuned in Matlab/Simulink with the PID tuner tool, in discrete time (*ts* = 0.002*s*). The values of PID controller gains obtained after tuning in Matlab are presented in the following Table 2.

**Table 2:** PID controller gains.

**Gain**	**K_**p**_**	**K_**d**_**	**K_**i**_**
Value	0.2	0.0005	0.002

**Figure 9:**
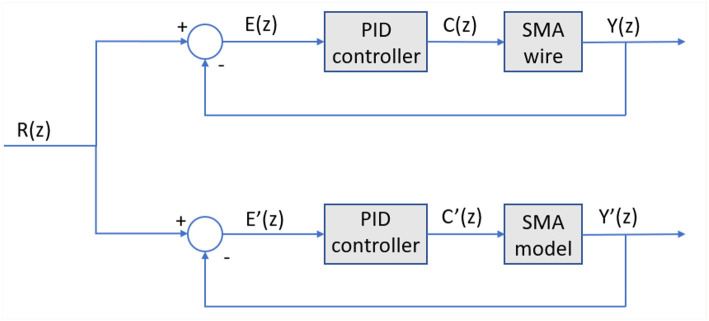
Control algorithm test for the real and simulated model of the SMA wire.

The proposed scheme to evaluate the model performance is presented in Figure 9. The SMA wire and the SMA wire model was tested in parallel and with the same PID controller (with the same gains). For the two controller scheme, the reference is the same and the outputs (final position) have been compared.

**Figure 10:**
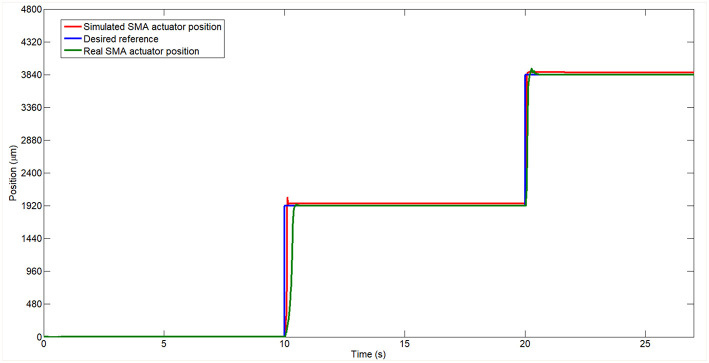
Real and simulated model of the SMA wire controlled in position tracking a step reference.

In Figure 9, *R*(*z*) represent the desired reference, E(k) represent the error (the difference between the desired position *R*(*z*) and the currently position of the SMA wire *Y*(*z*)) and the *C*(*z*) represent the signal generated by the PID controller. *E*′(*z*), *Y*′(*z*)) and *C*′(*z*) represent the error, currently position and controller signal of the SMA model scheme. The output signals (position) for the two systems when the desired reference is a step signal (*Y*(*z*) and *Y*′(*z*)) have been acquired and can be seen in Figure 10.

In Figure 10 the two systems follow the same reference signal represented by a stepped signal. The two responses are very similar and track the reference with accuracy. In the case of the real system, a slower displacement in transient stage can be observed in the first step compared with the simulated one. This corresponds to the martensite phase of the SMA wire, which presents a slower strain change with the temperature changes. In the second step, the output of the SMA wire is very similar with the model of the SMA wire but presents a little less overshoot compared with the SMA wire model.

**Figure 11:**
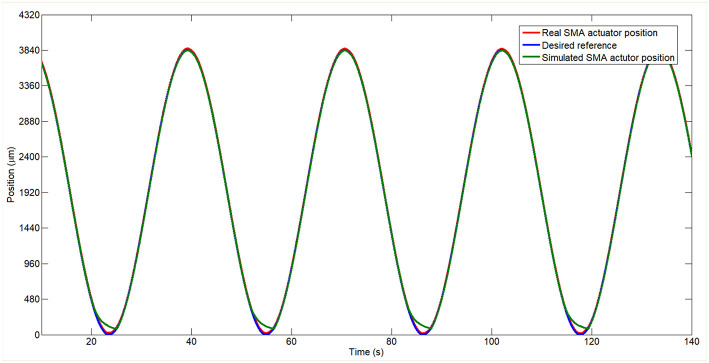
Real and simulated model of the SMA wire controlled in position tracking a sine reference.

The response of the SMA wire and the model of the SMA wire tracking a sine wave signal in position can be seen in the Figure 11. In this case, the behavior of the simulated SMA wire is very similar with the behavior of the real SMA wire. The biggest difference is in the cooling stage. The error between the two behaviors being of the order of μ*m*.

## 5. Conclusions

SMA actuators represent a suitable technology of actuation for the soft robotic devices, among others. This paper presents the methodology, development and the results of a new approach for the SMA wire modeling, which is a black box model based on Hammerstein–Wiener model. To achieve successful model implementation, the SMA wire was modeled separately in the two phases of transformation, with two different models: one model which characterizes the transformation from electrical energy to thermal energy and another which models the thermal energy to mechanical work. In the final model, SMA wire model is represented by these two models connected in series.

The identified model was introduced in a control algorithm where a PID controller was tuned. In the same time and with the same PID controller, the simulated SMA wire was compared with the real SMA wire in the test bench. The two systems present a similar behavior in the heating stage but present differences in the cooling stage, where the identification of the wire behavior was not possible due to the lack of the input signal.

The final SMA model can be used to test and tune different control algorithms before they are implemented in a real SMA wire or real systems actuated with SMA based actuators.

## Data Availability

The datasets generated for this study are available on request to the corresponding author.

## Author Contributions

DB and LM has overseen the project administration and funding acquisition. DC developed the SMA model and carried out the experiments. LM has collaborated in experiments and supervised the research. DC and DB wrote the manuscript. All the authors read and approved the final manuscript.

### Conflict of Interest Statement

The authors declare that the research was conducted in the absence of any commercial or financial relationships that could be construed as a potential conflict of interest.

## References

[B1] amsAG (2019). Nse-5310 Linear Sensor. Available online at: https://ams.com/nse-5310 (accessed February 1, 2019).

[B2] Bai E.-W. Cai Z. Dudley-Javorosk S. Shields R. K. (2009). Identification of a modified wiener–hammerstein system and its application in electrically stimulated paralyzed skeletal muscle modeling. Automatica 45, 736–743. 10.1016/j.automatica.2008.09.023 23467426 PMC3586551

[B3] Balestrino A. Landi A. Ould-Zmirli M. Sani L. (2001). Automatic nonlinear auto-tuning method for hammerstein modeling of electrical drives. IEEE Trans. Ind. Electron. 48, 645–655. 10.1109/41.925592

[B4] Butera F. Coda A. Vergani G. SpA S. G. (2007). Shape memory actuators for automotive applications, in Nanotec IT Newsletter (Roma: AIRI/Nanotec IT), 12–16.

[B5] Caballero A. F. Copaci D. S. Peciña Á. V. Rojas D. B. Lorente L. M. (2016). Sistema avanzado de protipado rápido para control en la educación en ingeniería para grupos multidisciplinares. Rev. Iberoam. Autom. Inform. Ind. RIAI 13, 350–362. 10.1016/j.riai.2016.05.004

[B6] Chau E. Friend C. Allen D. Hora J. Webster J. (2006). A technical and economic appraisal of shape memory alloys for aerospace applications. Mater. Sci. Eng. A 438, 589–592. 10.1016/j.msea.2006.02.201

[B7] Copaci D. Cano E. Moreno L. Blanco D. (2017). New design of a soft robotics wearable elbow exoskeleton based on shape memory alloy wire actuators. Appl. Bionics Biomech. 2017:1605101. 10.1155/2017/1605101 29104424 PMC5605786

[B8] Copaci D. Flores A. Villoslada A. Blanco D. (2015). Modelado y simulación de actuadores sma con carga variable, in Proceedings of the XXXVI Jornadas de Automática (Bilbao), 2–4.

[B9] Copaci D-S. Flores-Caballero A. Monar F. M. Blanco D. (2014). Modelado de motores usm para robotica de rehabilitacion. J. Autom. 35, 575–580.

[B10] Cui D. Song G. Li H. (2010). Modeling of the electrical resistance of shape memory alloy wires. Smart Mater. Struct. 19:055019 10.1088/0964-1726/19/5/055019

[B11] Dynalloy I. M. O. D. A. (2018). Technical Characteristics of Flexinol. Available online at: http://www.dynalloy.com/tech_data_wire.php (accessed May 1, 2018).

[B12] Eskinat E. Johnson S. H. Luyben W. L. (1991). Use of hammerstein models in identification of nonlinear systems. AIChE J. 37, 255–268. 10.1002/aic.690370211

[B13] Hartl D. J. Lagoudas D. C. (2007). Aerospace applications of shape memory alloys. Proc. Inst. Mech. Eng. G J. Aerosp. Eng. 221, 535–552. 10.1243/09544100JAERO211

[B14] Hughes D. Wen J. T. (1997). Preisach modeling of piezoceramic and shape memory alloy hysteresis. Smart Mater. Struct. 6:287 10.1088/0964-1726/6/3/007

[B15] Hunt K. J. Munih M. Donaldson N. D. N. Barr F. M. (1998). Investigation of the hammerstein hypothesis in the modeling of electrically stimulated muscle. IEEE Trans. Biomed. Eng. 45, 998–1009. 10.1109/10.704868 9691574

[B16] Hunter I. W. Korenberg M. J. (1986). The identification of nonlinear biological systems: Wiener and hammerstein cascade models. Biol. Cybern. 55, 135–144. 3801534 10.1007/BF00341929

[B17] Iadicola M. A. Shaw J. A. (2004). Rate and thermal sensitivities of unstable transformation behavior in a shape memory alloy. Int. J. Plast. 20, 577–605. 10.1016/S0749-6419(03)00040-8

[B18] Jani J. M. Leary M. Subic A. Gibson M. A. (2014). A review of shape memory alloy research, applications and opportunities. Mater. Des. 56, 1078–1113. 10.1016/j.matdes.2013.11.084

[B19] Le F. Markovsky I. Freeman C. T. Rogers E. (2012). Recursive identification of hammerstein systems with application to electrically stimulated muscle. Control Eng. Pract. 20, 386–396. 10.1016/j.conengprac.2011.08.001

[B20] Lennart L. (1999). System Identification: Theory for the User. Upper Saddle River, NJ: PTR Prentice Hall, 1–14.

[B21] Martín Clemente A. I. (2013). Modelado y control de sistemas no lineales de tipo SMA (Ph.D. Thesis). Universidad Carlos III de Madrid, Leganés, Madrid, Spain.

[B22] Matlab (2019). System Identification Toolbox. Available online at: https://www.mathworks.com/products/sysid.html (accessed February 13, 2019).

[B23] Melexis (2019). Mlx90614. Available online at: https://www.melexis.com/en/product/mlx90614/digital-plug-playinfrared-thermometer-to-can (accessed February 1, 2019).

[B24] Morgan N. (2004). Medical shape memory alloy applications–the market and its products. Mater. Sci. Eng. A 378, 16–23. 10.1016/j.msea.2003.10.326

[B25] Narendra K. Gallman P. (1966). An iterative method for the identification of nonlinear systems using a hammerstein model. IEEE Trans. Autom. Control 11, 546–550. 10.1109/TAC.1966.1098387

[B26] Novák V. Šittner P. Dayananda G. Braz-Fernandes F. Mahesh K. (2008). Electric resistance variation of niti shape memory alloy wires in thermomechanical tests: experiments and simulation. Mater. Sci. Eng. A 481, 127–133. 10.1016/j.msea.2007.02.162

[B27] Saes (2018). Saes Group. Available online at: https://www.saesgetters.com/products-functions/products/shapememory-alloys-nitinol (accessed May 1, 2018).

[B28] Song G. Chaudhry V. Batur C. (2003). Precision tracking control of shape memory alloy actuators using neural networks and a sliding-mode based robust controller. Smart Mater. Struct. 12:223 10.1088/0964-1726/12/2/310

[B29] Sreekumar M. Nagarajan T. Singaperumal M. Zoppi M. Molfino R. (2007). Critical review of current trends in shape memory alloy actuators for intelligent robots. Ind. Robot. Int. J. 34, 285–294. 10.1108/01439910710749609

[B30] Stoeckel D. (1990). Shape memory actuators for automotive applications. Mater. Des. 11, 302–307. 10.1016/0261-3069(90)90013-A

[B31] Sung S. W. (2002). System identification method for Hammerstein processes. Ind. Eng. Chem. Res. 41, 4295–4302. 10.1021/ie0109206

[B32] Velázquez R. Pissaloux E. E. (2012). Modelling and temperature control of shape memory alloys with fast electrical heating. Int. J. Mech. Control 13, 1–8.

[B33] Wang J. Sano A. Chen T. Huang B. (2007). Blind Hammerstein identification for MR Damper modeling, in 2007 American Control Conference (New York, NY: IEEE), 2277–2282.

[B34] Wiener N. (1942). Response of a Non-linear Device to Noise. Technical report, Massachusetts Institute of Technology, Cambridge Radiation Laboratory, Cambridge, MA.

[B35] Zhang X.-l. Tan Y.-h. (2008). Modelling of ultrasonic motor with dead-zone based on Hammerstein model structure. J. Zhejiang Univ. Sci. A 9, 58–64. 10.1631/jzus.A071146

